# A novel intradermal combination vaccine for PCV2 and *Mycoplasma hyopneumoniae* protection in swine: its use with Lawsonia *intracellularis* and PRRSV vaccines

**DOI:** 10.1186/s40813-025-00426-9

**Published:** 2025-03-13

**Authors:** Basav N. Hangalapura, Maarten Witvliet, Antonius A.C. Jacobs, Ruud P.A.M. Segers

**Affiliations:** MSD Animal Health, PO Box 31, Boxmeer, 5830 AA The Netherlands

**Keywords:** Porcilis^®^ PCV M Hyo ID vaccine, *Lawsonia intracellularis*, Porcine reproductive and respiratory syndrome, Combination vaccine, Swine vaccines, Intradermal vaccination

## Abstract

The combined, intradermal application of multiple vaccines against key diseases in swine offers many benefits, including reduced time and labor costs, and improved animal welfare due to fewer injections and manipulations. This study investigated the efficacy of a newly developed intradermal combination vaccine for *Porcine circovirus* 2 (PCV2) and *Mycoplasma hyopneumoniae* (M hyo) (PCV M Hyo ID vaccine) in swine. The vaccine was evaluated for its efficacy against PCV2 and M hyo infection and its concurrent mixed use with *Lawsonia intracellularis* (LI) and non-mixed use with Porcine reproductive and respiratory syndrome virus (PRRSV) vaccines. The findings demonstrated that the PCV M Hyo ID combination vaccine is efficacious against PCV2 and M hyo infection. Furthermore, the new PCV M Hyo ID combination vaccine can also be administered simultaneously and at the same anatomical location after mixing with LI ID vaccine, and next to PRRS ID vaccine, to efficiently protect pigs from all four major diseases in swine. The efficacy with the combination of vaccines was equivalent to that of the single vaccines.

## Introduction

Porcine circovirus 2 (PCV2), *Mycoplasma hyopneumoniae* (M hyo), porcine reproductive and respiratory syndrome virus (PRRSV), and *Lawsonia intracellularis* (*LI*) are among the top four highly prevalent pathogens that pose significant health, welfare and economic threat to the swine industry at large [1, 2, 3, 4].

PCV2 is a single-stranded DNA virus that widely infects pigs, causing several syndromes, collectively called as Porcine Circovirus Diseases [5, 6], and is the main causative agent of PCV2 systemic disease. PCV2 infection in pigs causes lymphocyte depletion with granulomatous inflammation of lymphoid tissues, leading to weakened immune function and increased vulnerability to other infections.

M hyo is a cell wall deficient bacterial pathogen, that attaches to the ciliated epithelium in the trachea, bronchi, and bronchioles, leading to damage to the mucosal clearance system [7]. Additionally, it suppresses immune function and increases the susceptibility of pigs to other respiratory infections [8]. It is the main cause of enzootic pneumonia, a chronic respiratory disease in pigs, and a primary contributor to porcine respiratory disease complex [9]. Infections with M hyo are widespread in swine-producing regions, leading to economic losses due to increased medication use and reduced pig performance due to the disease [10].

PRRSV is a single-stranded positive-sense RNA virus that leads to reproductive failure in sows and respiratory disorders in pigs of all ages [11, 12]. PRRSV first emerged in North America in 1987 [13] and in Europe in 1990 [14]. PRRSV is categorized into two species: Betaarterivirus suid 1 (formerly known as PRRSV-1 or European-type PRRSV) and Betaarterivirus suid 2 (formerly known as PRRSV-2 or North American-type PRRSV) [15].

PCV2, M hyo and PRRSV are the most clinically important pathogens in the Porcine Respiratory Disease Complex, which is a multifactorial and complex disease characterized by respiratory disorders and poor growth in growing and finishing pigs [16, 17].

LI is an obligate intracellular bacterium that is responsible for causing Porcine proliferative enteropathy (PPE). This condition is manifested by the hyperplasia of crypt enterocytes in the ileum and colon, leading to various clinical signs. PPE presents itself in two primary clinical forms: acute proliferative hemorrhagic enteropathy (PHE) and chronic porcine intestinal adenomatosis (PIA) [18]. The acute form results in hemorrhagic diarrhea and sudden death, predominantly affecting young pigs between 4 and 12 months of age. On the other hand, the chronic form is characterized by diarrhea or subclinical infection, leading to weight and productivity losses, particularly in pigs aged 6 to 20 weeks [19].

Vaccination, along with good farm management practices, has been proven to be effective in preventing or managing diseases caused by PCV2, M hyo, PRRSV and LI on swine farms. Since there is no single combination vaccine that effectively targets all four major pathogens, swine practitioners and producers often rely on individual vaccines, PCV2 and M hyo combination vaccines or those that can be mixed at the time of administration [20].

In order to enhance animal welfare and minimize pain and stress associated with multiple vaccinations, reduce costs, prevent needle stick injuries to administrators, iatrogenic infection transmission, and broken needles in meat products, many practitioners and producers prefer to use a single-dose, combination vaccine against major pathogens that can be applied in a single dose using needle-free devices. Developing such a combination vaccine, which meets all the required safety, efficacy, shelf-life, costs, etc. is still challenging. As a first step, we recently demonstrated that four intradermal vaccines, Porcilis PCV ID, Porcilis M Hyo ID ONCE, Porcilis Lawsonia ID, and Porcilis PRRS ID can be administered at the same time at the same anatomical site using needle-free vaccination devices with a twin-nozzle IDAL^®^ 3G Twin (This instrument allows vaccines in two separate vials to be applied simultaneously, Henke-Sass Wolf, Germany) and a single-nozzle IDAL^®^ 3G Mono (Henke-Sass Wolf, Germany) (Horsington et al., [21]. PCV ID is a vaccine that contains PCV2 virus-like particles (VLP) in a ready-to-use form, including the adjuvant X-Solve12 [22]. M Hyo ID ONCE is a vaccine comprising inactivated whole M hyo bacterium in X-Solve50 adjuvant [23]. Lawsonia ID is a freeze-dried vaccine containing inactivated LI, which is reconstituted before use in either X-Solve12 or PCV ID vaccine [21, 24]. PRRSV vaccine is a live-attenuated PRRSV1 vaccine that is freeze-dried and is reconstituted using the adjuvant Diluvac Forte before its application, either intradermally or intramuscularly [25].

In this study, we present a newly developed ready-to-use combination vaccine, Porcilis^®^ PCV M Hyo ID, containing inactivated PCV2 and M hyo antigens for intradermal application. This vaccine is designed to enhance user convenience, reduce vaccination costs, and minimize the number of injections administered to piglets. Additionally, it aims to lower the environmental carbon footprint of the vaccine by decreasing medical waste, reducing packaging costs, and minimizing the energy and costs associated with transportation and storage.

The primary objective of this study was to evaluate the efficacy of a newly developed combination vaccine PCV M Hyo ID, against PCV2 and M hyo infection. The secondary objective was to evaluate whether this new combination vaccine can be mixed with Lawsonia ID and applied simultaneously next to PRRS ID intradermal vaccine, at the same time and at the same side of the neck to protect pigs against all four major pig diseases in a single handling. Efficacy was demonstrated through vaccination-challenge studies with the individual pathogens.

## Materials and methods

### Animals

Approximately three weeks old male and female piglets of several litters were used for each study. All piglets were vaccinated prior to weaning.

### Vaccines

The vaccines tested in this study were PCV M Hyo ID, PRRS, and Lawsonia ID. The vaccines were administered intradermally using the IDAL^®^ 3G Mono or Twin. IDAL Twin is an intradermal vaccination device with two injector heads that are 3 cm apart, enabling the simultaneous delivery of two vaccines in a volume of 0.2 ml each, with a single handling. The manufacturer’s recommended dosage of each vaccine was administered singly or in combination, with the exception of PRRS vaccine. In the case of PRRS vaccine, the dose administered was 1 × 10^4^ TCID_50_ in the PRRSV challenge study and 1 × 10^6^ TCID_50_ in the other studies. The lyophilized PRRS was reconstituted with Diluvac Forte and the lyophilized Lawsonia ID was reconstituted with either PCV M Hyo ID, or X-Solve12.

### PCV2 study design and sample analysis

Piglets of Landrace x Large white breed that tested negative for PCV2 DNA in their blood and had PCV2 maternal antibody titers lower than 6 log_2_ [26] were divided into three groups, each consisting of 15 piglets. At around three weeks of age, the piglets in the first group (PCV-G1) were vaccinated intradermally with PCV M Hyo ID mixed with Lawsonia ID, next to PRRS vaccine, using the IDAL^®^ 3G Twin. The second group (PCV-G2) received the PCV M Hyo ID vaccine only. The third group (PCV-G3) did not receive any vaccination and served as the control group for the challenge.

At two weeks post-vaccination (5 weeks of age, study day 14), all the piglets were exposed to a wild-type PCV2b challenge virus strain I-12/11 (an isolate from a Dutch pig farm), administered intranasally at a dosage of 5.8 log_10_ TCID_50_ in a total volume of 6 mL (3mL per nostril). The piglets were monitored daily for any clinical signs, and blood samples and fecal swabs were collected throughout the study. Three weeks after the challenge, all the animals were humanely euthanized using electric stunning (> 250 V, > 1.3 A) followed by exsanguination, and samples were taken from the inguinal lymph node, mesenteric lymph node, tonsil, and lung to detect PCV2 nucleic acid.

The serum was isolated from blood samples and stored at -20°C until tested. The anti-PCV2 antibodies in sera were measured using ELISA, as per the description in a previous study [26]. DNA from the serum, rectal swabs, and tissue homogenates was extracted using a commercial kit (Roche, Magnapure 96 with DNA/viral NA SV kit), and the PCV2 DNA was quantified using custom made qPCR with primers (F1: 5’- GTA **A**CG GGA GTG GTA GGA GAA − 3’, F2: 5’- GTA **G**CG GGA GTG GTA GGA GAA − 3’, R1: 5’- GCC ACA GCC CT**A** ACC TAT GAC − 3’. R2: 5’- GCC ACA GCC CT**C** ACC TAT GAC − 3’) and dual hydrolysis probe (5’- 6FAM- ATG TAA ACT ACT CCT CCC GCC ATA CCA TA -BHQ1-3’) specific for PCV2-ORF2. The resulting cycle numbers were correlated to a set of samples containing known amounts of PCV2-ORF2-containing plasmid, and the results were expressed as log_10_ copies/µl of extracted DNA. Values lower than 1.00 log_10_ copies/µl were considered negative and treated as 0.00 log_10_ copies/µl for calculation.

### PRRSV study design and sample analysis

Piglets of Duroc x York breed, which tested negative for PRRSV viral RNA in their blood and lacked PRRSV antibodies, were assigned to three treatment groups, with each group containing 15 piglets. Due to a human error, two piglets from the PRRS-G2 group were inadvertently excluded during the vaccination process.

At approximately three weeks of age (study day 0), piglets in the PRRS-G1 group were vaccinated similarly to the PCV-G1 group. The PRRS-G2 group received only the PRRS vaccine. The PRRS-G3 group was not vaccinated and served as the control group for the challenge. At four weeks post-vaccination (7 weeks of age, study day 28), all the piglets were exposed to a virulent PRRSV Type 1 field strain, Isolate 2 from a Dutch pig farm, applied intranasally at a dosage of 5.3 log_10_ TCID_50_ in a total volume of 2 mL (1mL per nostril). Throughout the study, the piglets were monitored daily for clinical signs, and their rectal temperatures were recorded from one day before the challenge until 14 days after the challenge (study day 27 to 42). The pigs were weighed on study day 27 and study day 56 (end of study), and the average daily weight gain (ADWG) was calculated for each individual animal and averaged by group. Blood samples were collected on study day 0, study day 27, and study day 56, and serum was isolated and stored at -20 °C until testing. Anti-PRRSV antibodies were assessed using an ELISA test kit (IDEXX PRRS X3) according to the manufacturer’s instructions. In this kit, an S/P value of ≥ 0.4 is considered positive. PRRSV in the serum was quantified by titration on porcine alveolar macrophage (PAM) cells. This involved the inoculation of PAMs with the test sample, which was serially diluted 10-fold (6 wells per dilution), and then incubated at 37 °C. PRRSV infection in the cells was observed after 6–7 days by checking for cytopathic effects (CPE). Titers were calculated using the Spearman-Kärber method and expressed as log_10_ TCID_50_/ml. At study day 56, all the animals were humanely euthanized using electric stunning (> 250 V, > 1.3 A) followed by exsanguination.

### M hyo study design and sample analysis

Piglets of Landrace x Large white breed with either no antibody titers against M hyo, PRRSV and LI or low antibody titers against PCV2 (< 6 log_2_), were divided into 2 treatment groups, each consisting of 20 piglets. At approximately three weeks of age, the pigs in the M hyo-G1 group were vaccinated intradermally with PCV M Hyo ID mixed with Lawsonia ID, alongside the non-mixed PRRS, using the IDAL 3G Twin. M hyo-G2 group was vaccinated with only PRRS vaccine and served as the group for challenge control.

Four weeks after vaccination (7 weeks of age), all the animals were infected with a virulent Danish M hyo field isolate, delivered via the intratracheal route for two consecutive days with 10 ml of pure culture (10^9^ and 10^8^ color changing units/ml for the first and second day of challenge, respectively). Blood samples were collected before vaccination, just before the infection, and at necropsy. The serum was examined for antibodies using the IDEXX^®^ M hyo Ab test and the IDEXX^®^ PRRS X3 Ab test as per the manufacturer’s instructions. Three weeks post-challenge infection, all the animals were humanely euthanized using electric stunning (> 250 V, > 1.3 A) followed by exsanguination and examined for lung lesions. The scoring of lung lesions was carried out in accordance with Ph. Eur. monograph 2448.

### Lawsonia study design and sample analysis

Piglets of Duroc x York breed with either no or low (< 5.1 log_2_) maternally-derived antibodies against LI were allotted into 3 treatment groups of 25 piglets each. At around three weeks of age, animals in Laws-G1 were vaccinated intradermally with PCV M Hyo ID mixed with Lawsonia ID vaccine next to PRRS vaccine (non-mixed), using the IDAL^®^ 3G Twin. The Laws-G2 group was vaccinated with only Lawsonia ID vaccine. Laws-G3 was not vaccinated and served as a challenge control. At four weeks post vaccination (7 weeks of age, SD28) all animals were orally challenged with 20 ml homogenized LI infected intestinal mucosa.

Following the challenge, the pigs were monitored daily for clinical signs of LI infection and scored according to the protocol described previously [24]. In this challenge model, clinical signs become apparent in the third week after challenge. The daily clinical scores from 13 to 21 days post-challenge were totaled and averaged by group.

Weight measurements were taken one day before the challenge and on days 6, 13, and 20 after the challenge and the average daily weight gain (ADWG) was calculated for each individual animal and averaged by group.

Three weeks post challenge (SD49), the pigs were humanely euthanizedusing electric stunning (> 250 V, > 1.3 A) followed by exsanguination and a post-mortem examination was carried out. During necropsy the intestines, particularly the ileum (i.e., the distal 50 cm of the small intestine), were examined for lesions indicative of PPE. A fecal sample (from the rectum) and an ileum mucosa sample (5 cm above the ileo-caecal junction) were collected from each animal for testing using a LI specific qPCR. Additionally, an ileum sample was collected, fixed in 4% buffered formalin, paraffin-embedded and processed into slides, which were then stained with Haematoxylin-Eosin (HE stain) and with an immunohistochemical (IHC) stain using an anti-LI monoclonal antibody (IHC stain) and were examined microscopically.

The ileum mucosa was macroscopically scored as described previously [24] and the percentage of the ileum affected was estimated as follows: the length of the affected part of the ileum was divided by the length of the ileum and multiplied by 100. The total ileum lesion score was calculated by multiplication of the ileum mucosa score and the percentage of ileum affected. The average total ileum lesion score was calculated for each treatment group.

The histological scoring (HE score and the IHC score) was performed as described previously [24]. The total histological score was calculated by multiplication of the HE score and the IHC score. The average total histological score was calculated for each group.

Sera was isolated from blood samples collected on SD0, 28 and 49 and stored at -20 °C until tested. Serum samples were tested in an LI antibody ELISA, as described previously [24]. DNA was extracted from 0.2 g feces or mucosa sample using a commercial kit (Roche, Magnapure 96 with DNA/viral NA SV kit) and LI DNA quantified by qPCR, as described previously [24].

### Statistical analysis

Statistical analysis was conducted using SAS (SAS Institute Inc. Cary NC, USA) as described below. Tests were two-sided, using a significance level of 5%.

#### PCV2

PCV2 antibody titers at time of vaccination were analyzed by Analysis of Variance (ANOVA). PCV2 titers at time of challenge were analyzed by ANOVA with titer at vaccination as a covariate. PCV2 titers after challenge were analyzed by ANOVA for repeated measurements. For viral load in serum and rectal swabs, the Area-Under-the-Curve (AUC) of the qPCR results after challenge was calculated by the trapezoidal rule and analyzed by the Kruskal-Wallis test. qPCR results in each lymphoid tissue/organ were analyzed by the Kruskal-Wallis test.

#### PRRSV

Body temperature after challenge was statistically analyzed by ANCOVA for repeated measurements using the average pre-challenge temperature as covariate. ADWG was statistically analyzed by ANCOVA using the pre-challenge weight as a covariate. PRRSV viremia after challenge was analyzed by calculation of the AUC. A virus titer of < 1.15 log_10_ was set to a titer of 0.00 log_10_ for use in the calculations. The AUC was calculated by means of the linear trapezoidal rule and statistically analyzed by ANOVA. Additionally, post challenge viremia data was converted into a positive / negative outcome over time for each piglet and statistically analyzed by Generalized Estimating Equations (GEE), accounting for the correlation in the repeated measurements on an animal. The *p*-value was based on the GEE empirical standard error. As part of this the odds ratio (OR) with its 95% confidence interval was calculated. The OR provides a relative measure of the effect of vaccination in reducing the incidence of post-challenge viremia.

#### M hyo

Lung lesion scores were analyzed using a Wilcoxon rank-sum test.

#### LI

Antibody titers were evaluated using descriptive statistics. The average values were plotted with the standard deviation.

The diarrhea scores between days 13 and 21 were statistically analyzed by a cumulative logit model [27] accounting for the correlation in the repeated measurements using GEE with *p*-values based on empirical standard error.

The ADWG in this period (days 13 to 20) was calculated and statistically analyzed by ANCOVA using the weight at day 13 as covariate and using Tukey’s post-hoc test to compare groups.

Quantitative PCR data from feces and ileum mucosa samples were log_10_ transformed (after adding 1 to avoid zeros) and expressed in log_10_ pg DNA/µl. The average values were plotted with the 95% confidence interval where no overlap indicates statistical significance. In addition, the AUC was calculated by the linear trapezoidal rule as a measure of total shedding over time. The AUC of the qPCR data of feces, the feces qPCR data on day 21 and the qPCR data of the ileum mucosa on day 21 were statistically analyzed by ANOVA using Tukey’s post-hoc test to compare groups.

The macroscopic total ileum lesion score and the total histology score were statistically analyzed by a cumulative logit model with *p*-values based on Likelihood-Ratio. The odds ratio was defined here as the odds on having lower classes in the vaccine group relative to that in the control group. The mortality was evaluated by a generalized linear mixed model for binomials using a logit link with treatment as fixed effect.

## Results

### Protection against PCV2 challenge

**Fig. 1:**
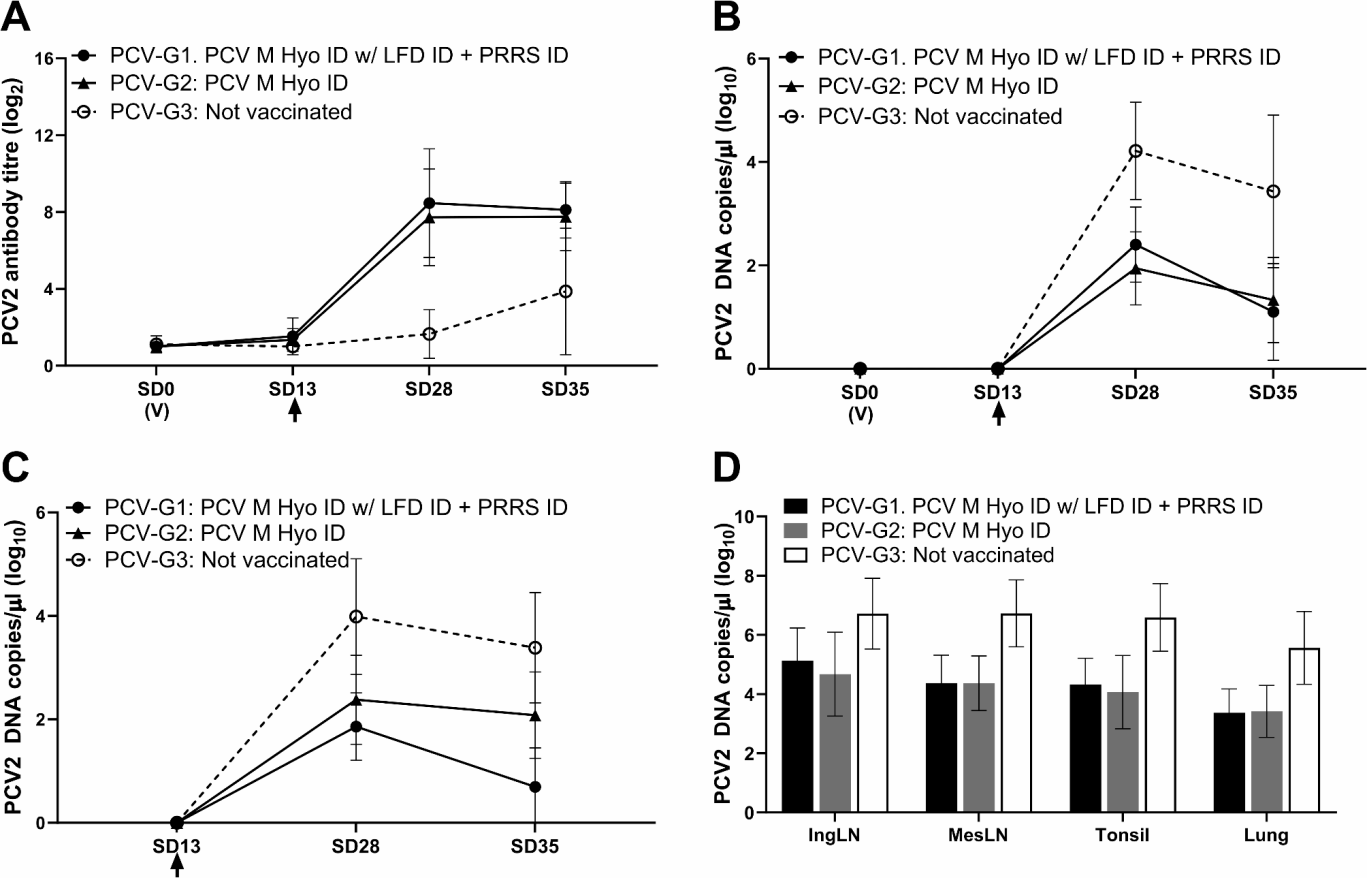
PCV2 challenge study results. (**A**) mean antibody response in PCV2 ELISA; (**B**) PCV2 viraemia (mean DNA load in serum); (**C**) mean PCV2 DNA load in rectal swab samples; (**D**) mean PCV2 DNA load in tissue homogenates. (V) indicates time of vaccination. Arrow indicates challenge. Error bars show standard deviation

During the study, none of the pigs showed any clinical signs related to either vaccination or challenge exposure. There was one death, unrelated to vaccination or challenge. Pigs receiving the combination vaccination PCV M Hyo ID mixed with Lawsonia ID, next to PRRS, using the IDAL^®^ 3G Twin (PCV-G1) or PCV M Hyo ID (PCV-G2) alone had similar PCV antibody responses, which were significantly higher than the non-vaccinated control group (PCV-G3) (both *p* < 0.0001) (Fig. 1A). Both PCV-G1 and PCV-G2 exhibited a significant reduction in viremia (both *p* < 0.0001; Fig. 1B), viral load in rectal swabs (both *p* < 0.001; Fig. 1C), and viral load in all tissues tested (all *p* < 0.01; Fig. 1D), when compared to PCV-G3.

### Protection against M hyo challenge

None of the pigs displayed clinical signs related to either vaccination or challenge exposure during the study, and there were no deaths. After the M hyo challenge, vaccinated pigs showed a strong anamnestic response (data not shown). Three weeks post M hyo challenge, examination of lung lesions showed a significant reduction (*p* < 0.01) in vaccinated pigs when compared to the control group (Table 1).

**Table 1:** Median lung lesion score per group and reduction in median compared to control group G3

Group	Treatment	Lung Lesion Score	
		Median*	Reduction
M hyo-G1	PCV M Hyo ID w/LFD ID + PRRS ID	1.6^a^	58%
M hyo-G2	PRRS ID	3.8^b^	-

* Scores with different superscript are significantly different from each other (*p* < 0.01)

### Protection against PRRSV challenge

**Fig. 2:**
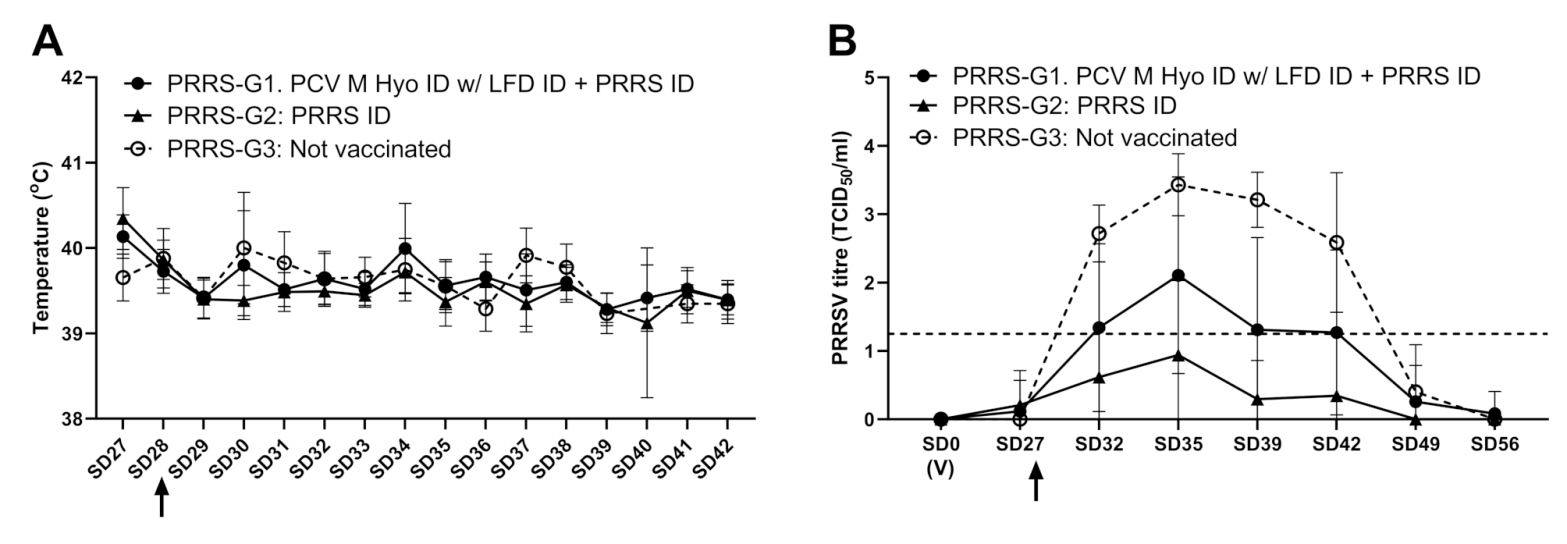
PRRSV challenge study results. **A** – mean rectal temperatures during the challenge period; **B** – PRRSV viraemia (mean virus titre in serum titrated on PAM cells, dotted horizontal line indicates assay cut-off). (V) indicates time of vaccination. Arrow indicates challenge. Error bars show standard deviation

None of the pigs displayed clinical signs related to vaccination. Some animals in all groups showed intermittent pyrexia (> 41.5 °C) in the post-challenge period, but the mean rectal temperature in all groups was below the pyrexia level (Fig. 2A). There were no deaths during the study. On the day of challenge, all pigs in the PRRS group (PRRS-G2) had seroconverted, whereas 67% in the combination group (PRRS-G1) had seroconverted (Table 2). Both G1 and G2 were significantly protected from PRRSV1 challenge infection when compared to the control group (PRRS-G3) (Fig. 2B). PRRS-G2 were protected better against viremia when compared to the combination group (PRRS-G1) (*p* < 0.01, Fig. 2B; Table 2). The vaccinated pigs (PRRS-G1 and G2) had similar average daily weight gain, which for both groups were significantly higher than the non-vaccinated pigs (for both *p* < 0.0001; Table 2).

**Table 2:** Seropositivity to PRRSV pre-vaccination (SD0), pre-challenge (SD27) and end of study (SD56), Area-Under-the-curve (AUC), and average daily weight gain post-challenge (SD27 – SD56)

Group	% seropositive to PRRSV antibodies			PRRSV Viraemia	ADWG (g) [st.dev.]^*^
	SD0	SD27	SD56	Mean AUC^*^	
PRRS-G1: PCV M Hyo ID w/LFD ID + PRRS ID	0.0	67.0	100.0	26.1^a, b^ [± 19.5]	812^a^ [± 129]
PRRS-G2: PRRS ID	0.0	100.0	100.0	9.0^a^ [± 7.3]	859^a^ [± 86]
PRRS-G3: Not vaccinated	0.0	0.0	93.0	49.8^b^ [± 9.8]	655^b^ [± 61]

* Values with different superscripts are significantly different from each other (*p* < 0.0001)

### Protection against LI challenge

**Fig. 3:**
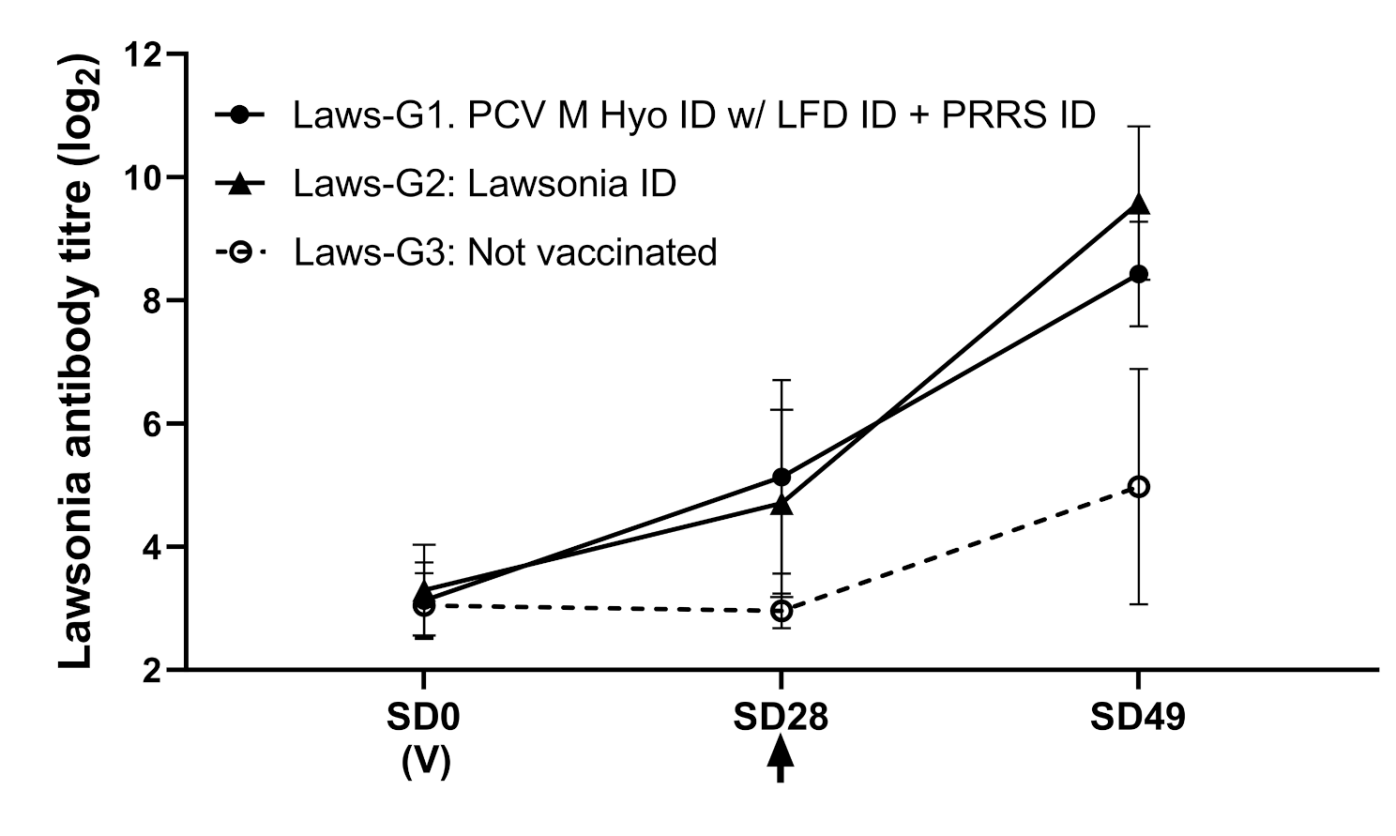
Antibody response to LI in ELISA. (V) indicates time of vaccination. Arrow indicates challenge. Error bars show standard deviation

On SD32, one pig in Laws-G3 was euthanized due to increasing locomotory and neurological signs (humane endpoint). Necropsy revealed fibrinous polyserositis involving right tarsus, abdominal cavity, and meninges. *Streptococcus suis* was isolated from tarsus and meninges. Pigs receiving the combination vaccination (Laws-G1) had similar LI antibody responses to those receiving Lawsonia ID alone (Fig. 3). Both Laws-G1 and Laws-G2 showed a significant increase in average daily weight gain and a significant reduction in diarrhea score, LI DNA load in feces, and ileum mucosa, macroscopic ileum score, and microscopic ileum score, compared to the non-vaccinated controls (Table 3).

**Table 3:** Post LI challenge results (mean +/- st.dev.)

Group	Mean diarrhoea score 13–21 dpc	ADWG g/day 13–20 dpc	qPCR faeces (mean log_10_ pg DNA/µl)		qPCR ileum mucosa 21 dpc (mean log_10_ pg DNA/µl)	Mean macroscopic ileum score 21 dpc	Mean microscopic ileum score (IHC) 21 dpc
			AUC	21 dpc			
Laws-G1: PCV M Hyo ID/LFD ID + PRRS ID	0.16 ± 0.62^a^	1108 ± 423^a^	0.14 ± 0.61^a^	0.04 ± 0.22^a^	0.01 ± 0.0^a^	0.4 ± 2^a^	0.1 ± 0.0^a^
Laws-G2: Lawsonia ID	0.08 ± 0.28^a^	1002 ± 303^a^	0.69 ± 1.38^a^	0.11 ± 0.40^a^	0.07 ± 0.21^a^	8.2 ± 26^a^	0.7 ± 1.9^a^
Laws-G3: Not vaccinated	2.00 ± 4.34^b^	668 ± 464^b^	2.90 ± 1.75^b^	1.18 ± 1.00^b^	0.43 ± 0.38^b^	108.5 ± 153^b^	4.1 ± 3.4^b^

^a^ significantly (*p* < 0.05) different to ^b^ within each category

## Discussion

The newly developed PCV M Hyo ID combination vaccine is efficacious against PCV2 and M hyo infections. Its efficacy was comparable to the separate administration of individual intradermal PCV2 and M hyo vaccines [21]. Additionally, this combination vaccine can be seamlessly combined with Lawsonia ID and administered concurrently with PRRS vaccine on the same side of the neck, offering comprehensive protection against all four major diseases in a single application. This development represents a significant advancement for the swine health management and offers several advantages, such as saving time and reducing labor costs, as well as improving animal welfare by minimizing injections and handling, and decreasing the energy footprint of the vaccines.

Previously, we demonstrated the effectiveness of intradermal vaccination against PCV2, PRRSV, M hyo and LI when using monovalent vaccines at the same anatomical location without compromising on the vaccine safety and efficacy [21]. To further enhance the user convenience, decrease the number of injections, and reduce the environmental impact of vaccines, a ready-to-use combination vaccine, PCV M Hyo ID, was developed. This study aimed to evaluate the efficacy of this new dual-action vaccine and its compatibility with Lawsonia ID and PRRS vaccines using the IDAL^®^ 3G Twin intradermal vaccinator to protect pigs against PCV2, M hyo, PRRSV and LI infections, all at the same anatomical site and without impacting vaccine efficacy. Challenge studies were conducted for each pathogen, comparing the protection provided by the combined intradermal vaccines with that of single vaccines (or with the PRRSV vaccine in the case of M hyo). The safety profiles of the vaccines were similar between single or combined vaccinations and in line with the specific characteristics of each vaccine (data not shown). Significant reduction in viremia and viral load was observed for PCV2 challenge in both the combination and single vaccine groups. In the PRRSV challenge, there was a significant reduction in viremia and an increase in ADWG in both the combination group and single vaccine group when compared to the control group. In contrast to the previous finding where no difference was observed [21], in this study single vaccine conferred a better protection against viremia when compared to the combination group. Most importantly, no difference in the increase in ADWG was observed between combination and single vaccination groups. Notably, in the M hyo challenge, the combination group showed a significant reduction in lung lesion score compared to the control group, and in the LI challenge, both the combination and single vaccine groups displayed significant increase in ADWG and a significant reduction in diarrhea score, LI DNA load in feces and ileum mucosa, macroscopic ileum score and microscopic ileum score.

PRRS is a modified live vaccine (MLV), and PRRSV is known to dampen the immune response. Infection with PRRSV or use of an MLV PRRSV vaccine was previously reported to reduce the efficacy of M hyo vaccines [28], however in contrast, with PCV ID, M Hyo ID, Lawsonia ID and porcine parvovirus vaccine, no interference by PRRS vaccination was observed [29, 30, 21]. In this study, no significant differences in clinical signs and clinical protection against all challenges, except viremia in PRRSV challenge, were observed between the groups receiving the combination of vaccines, and the groups receiving the single vaccines, indicating no interference between the vaccines or negative effects on efficacy from the simultaneous administration.

In intensive swine farming, routine practices such as vaccinations, which involve handling and pain, can negatively impact pig immune status and overall health [31, 32, 33]. By reducing handling and vaccination moments and administering vaccines against all major pathogens intradermally, positive impacts on animal welfare can be achieved [33, 34]. The findings demonstrate that intradermal vaccination with a needle-free device (e.g., IDAL^®^ 3G or IDAL^®^ 3G Twin) allow easy and rapid vaccination of pigs with the added benefits of a needle-free, intradermal vaccination, improving convenience and animal well-being.

One of the key benefits of this combination vaccine formulation is the flexibility it allows in broader disease management. This study demonstrated that the PCV M hyo ID vaccine can be successfully mixed and administered with LI vaccine through the same intradermal route, providing further protection against one of the major gastrointestinal pathogens affecting swine. Moreover, the intradermal vaccine can be administered concurrently with PRRSV vaccine using a twin intradermal applicator. This capability allows for simultaneous vaccination against four major diseases: PCV2, M hyo, PRRSV and LI, significantly enhancing the scope of preventive measures achievable in a single handling session. Such a strategy not only streamlines the vaccination process but also minimizes stress and discomfort for the animals by reducing the number of handling events required, which has been previously associated with animal stress and potential impacts on immune response.

From an economic and environmental perspective, the introduction of this combined vaccine offers substantial benefits. The consolidation of vaccines translates to fewer individual doses, reducing production demands and the associated costs of manufacturing, packaging, storage and transportation. This can lead to a decrease in energy usage and overall carbon footprint, aligning with sustainable practices that are becoming increasingly valued in veterinary medicine and animal production industries.

In terms of practical application, this combined intradermal vaccine regimen is highly advantageous in swine production settings where labor efficiency and resource optimization are critical. The ability to administer vaccines for four distinct diseases in one handling session addresses the operational challenges faced by swine producers, particularly in large-scale operations where labor shortages and cost efficiency are ongoing concerns. The reduction in handling time per animal not only increases farm productivity but also supports more humane management practices by minimizing the stress associated with repeated handling.

Finally, the intradermal route itself is a promising avenue for vaccine administration in swine. Intradermal vaccination has been shown to produce robust immune responses while being minimally invasive, thus supporting enhanced compliance with welfare standards in livestock production. This method also aligns with consumer and regulatory expectations for the humane treatment of production animals, as it causes less discomfort compared to traditional intramuscular vaccination.

## Conclusions

In conclusion, the newly developed combination vaccine, PCV M Hyo ID, against PCV2 and M hyo infections, has exhibited comparable efficacy to that of the individual vaccines. Additionally, this combination vaccine can be seamlessly combined with Lawsonia ID and administered concurrently with PRRS intradermal vaccine on the same side of the neck, offering comprehensive protection against all four major diseases in a single application. This approach not only preserves vaccine efficacy but also offers economic, environmental, and welfare advantages, supporting a more sustainable and humane approach to swine health management. Future studies should continue to explore the possibility to combine all the four antigens in a single vaccine with the possibility of further integration with additional vaccines to expand the protective benefits for swine health.

## Data Availability

Data can be made available upon reasonable request to the corresponding author.
